# Disruption of Network Synchrony and Cognitive Dysfunction After Traumatic Brain Injury

**DOI:** 10.3389/fnsys.2016.00043

**Published:** 2016-05-17

**Authors:** John A. Wolf, Paul F. Koch

**Affiliations:** ^1^Center for Brain Injury and Repair, Department of Neurosurgery, University of PennsylvaniaPhiladelphia, PA, USA; ^2^Corporal Michael J. Crescenz VA Medical CenterPhiladelphia, PA, USA

**Keywords:** TBI, concussion, axonal injury, synchrony, ensemble coding, oscillations, limbic system, hippocampus

## Abstract

Traumatic brain injury (TBI) is a heterogeneous disorder with many factors contributing to a spectrum of severity, leading to cognitive dysfunction that may last for many years after injury. Injury to axons in the white matter, which are preferentially vulnerable to biomechanical forces, is prevalent in many TBIs. Unlike focal injury to a discrete brain region, axonal injury is fundamentally an injury to the substrate by which networks of the brain communicate with one another. The brain is envisioned as a series of dynamic, interconnected networks that communicate via long axonal conduits termed the “connectome”. Ensembles of neurons communicate via these pathways and encode information within and between brain regions in ways that are timing dependent. Our central hypothesis is that traumatic injury to axons may disrupt the exquisite timing of neuronal communication within and between brain networks, and that this may underlie aspects of post-TBI cognitive dysfunction. With a better understanding of how highly interconnected networks of neurons communicate with one another in important cognitive regions such as the limbic system, and how disruption of this communication occurs during injury, we can identify new therapeutic targets to restore lost function. This requires the tools of systems neuroscience, including electrophysiological analysis of ensemble neuronal activity and circuitry changes in awake animals after TBI, as well as computational modeling of the effects of TBI on these networks. As more is revealed about how inter-regional neuronal interactions are disrupted, treatments directly targeting these dysfunctional pathways using neuromodulation can be developed.

## Introduction

Traumatic brain injury (TBI) can disrupt cognitive processing and memory function for many years post injury (Capruso and Levin, 1992; Ryan and Warden, 2003; Monti et al., 2013). TBI is a complex, heterogeneous disorder with many factors contributing to a spectrum of severity from concussion (mild TBI) to severe brain injury. Injuries across this spectrum may include damage to the white matter, where many axons of the brain coalesce into tracts that run throughout the brain (Johnson et al., 2013b). Diffuse axonal injury, detected histopathologically post mortem, is the hallmark neuropathology resulting from inertial brain injury (Adams et al., 1982; Gennarelli et al., 1982). Axonal injury can also result from ischemia, raised intracranial pressure, and other complications of diffuse brain injury. Injury to axons can lead to disruption in the channels, ionic homeostasis and myelination necessary for action potential propagation.

Unlike an injury to only a discrete brain region, axonal injury is fundamentally an injury to the substrate by which all networks in the brain communicate. There has recently been shift away from the brain as a “computer” analogy—a collection of discrete processors that shuttle packets of information between them in various stages of analysis. Rather, the brain is now depicted as a series of dynamic, interconnected networks with emergent properties that communicate via long axonal pathways grouped into white matter tracts (Alivisatos et al., 2012). This architecture has been termed the “connectome” due to its complexity and the connected network structure that it represents (Sporns et al., 2005; Van den Heuvel and Sporns, 2011). Neurons communicate via these pathways and encode information within and between brain regions in a number of ways, many of which are timing dependent. Our central hypothesis is that traumatic injury to axonal connections may disrupt the exquisite timing of neuronal communication within and between brain networks, and that this may underlie aspects of post-TBI cognitive dysfunction.

We will briefly review the evidence for axonal injury and memory and executive function impairment following TBI. We then describe some of the basic mechanisms of network level communication, how they depend on precise timing, and how failure of these mechanisms may lead to cognitive problems. As an example, we then focus our discussion on hippocampal network communication on multiple scales and present specific hypotheses regarding how this may be disrupted after TBI.

## Diffuse Axonal Injury is the Hallmark of Inertial Traumatic Brain Injury

A series of studies have demonstrated that inertial brain injury leads to mechanical injury to axons resulting in axonal pathology (Gennarelli et al., 1982; Graham et al., 1988; Adams et al., 1989; Margulies et al., 1990; Smith et al., 1997). Diffuse axonal injury is the hallmark multi-focal neuropathology of inertial injury, and is detected post mortem using antibodies against transported proteins that accumulate due to cytoskeletal damage or secondary disconnection (for review see Johnson et al., 2013b). More recently, other populations of injured axons have been defined, suggesting that those with transport disruptions may only be a portion of the total injured population (Johnson et al., 2016). Degenerative processes associated with axonal injury may be long-lasting, as it has recently been demonstrated in a chronic TBI human cohort that active axonal degeneration is occurring up to 18 years post injury (Johnson et al., 2013a).

While diffuse axonal injury is a common neuropathological consequence of moderate to severe injury, increasing data suggest that damage to axons may also be an important aspect of mild TBI, or concussion. Axons are preferentially vulnerable to biomechanical forces during inertial rotation, which are a primary or contributing factor to TBIs at every severity, including concussion (Holbourn, 1943, 1945; Strich, 1961; Adams et al., 1982; Gennarelli et al., 1982; Browne et al., 2011). Due to the anisotropy of white matter tracts and the viscoelastic properties of axons, they are selectively vulnerable to rapid mechanical forces such as shear and tensile strain during inertial injury (Smith and Meaney, 2000; Johnson et al., 2013b). The role of axonal injury and its prevalence in mild TBI is an active area of investigation, but different lines of evidence are converging to suggest its involvement. There are only a few post-mortem studies of mild TBI with small cohorts, but those that have been performed detected axonal injury in many cases (Blumbergs et al., 1989, 1994). Recent advances in imaging such as diffusion tensor imaging (DTI) have suggested that white matter abnormalities correlate with cognitive deficiencies in mild TBI (see below), although the correlation between DTI and axonal pathology remains to be determined (Miles et al., 2008; Wu et al., 2010; Jorge et al., 2012; Siman et al., 2013; Rabinowitz and Levin, 2014). One supporting factor is the existence of blood biomarkers, some of which are breakdown products from axonal injury, and that correlate with outcome and cognitive measures post concussion (Siman et al., 2013, 2015; Shahim et al., 2014).

## Memory and Executive Function are Persistent Cognitive Deficits After TBI

Hippocampal dependent memory impairment after TBI is perhaps one of the most commonly modeled aspects of clinical TBI. Persistent memory impairment after TBI, including after mild TBI, represents significant morbidity: 25% of soldiers suffering only a diffuse TBI returning from assignment have significant memory impairments (Hoge et al., 2008), and many civilians have persistent impairment decades after their injury (Draper and Ponsford, 2008; Monti et al., 2013). Interestingly, in one of the only reports that examined mild TBI pathology in humans after death from unrelated injuries, it was noted that the fornix, the major output tract of the hippocampus, had axonal pathology in all of the five cases examined (Blumbergs et al., 1994). The fact that memory impairment and axonal pathology in memory-relevant structures both occur in milder TBI cases without more severe focal injuries to the brain suggests that memory impairment after TBI may be due in part to communication disruption within and between brain regions as a result of axonal injury.

Executive function is also persistently impaired following TBI (McDowell et al., 1997; Draper and Ponsford, 2008). Patients with TBI have demonstrated deficits in working memory paradigms, including the n-back task (Sanchez-Carrion et al., 2008), a form of memory task requiring prefrontal executive function. Furthermore, severity of executive function deficits correlates with the degree of frontal white matter abnormalities in human DTI (Kinnunen et al., 2011). Interactions between the prefrontal cortex (PFC) and the hippocampus are known to be central to the integration of executive function and memory. DTI in humans suggests that limbic white matter pathways, including the cingulate bundle which connects the PFC and the hippocampus, are damaged following diffuse TBI (Niogi et al., 2008a,b; Spitz et al., 2013) and that this damage correlates with memory and attention impairment in humans (Niogi et al., 2008b). Finally, cognitive processing speed, which supports all executive and memory functions, has been demonstrated as a deficit post-injury (Draper and Ponsford, 2008), with hippocampal fornix axonal dysfunction suggested as one potential substrate (Fisher et al., 2000; Mathias et al., 2004; De Monte et al., 2006; Yallampalli et al., 2013).

## Network Dysfunction Following Axonal Injury

How might diffuse axonal injury following TBI contribute to the well-established deficits in memory and executive function? Answering this broad question is one major goal of TBI research and requires the formulation and testing of an extensive series of hypotheses that span multiple scales of organization, from subcellular changes in the cytoskeleton, to intrinsic properties of individual neurons, to the formation of local “neuronal ensembles” (see below), and to the function of regional and global neural networks (i.e., the connectome).

### Axonal Injury Disrupts Timing of Neuronal Signal Conduction

At the cellular and subcellular scale alterations in intrinsic neuronal properties and conduction delays following TBI have been examined using *ex vivo* brain slice preparations from rodent TBI models, or *in vitro* neuron culture preparations that have been mechanically injured. Axonal injury can lead to secondary changes in the channels, ionic homeostasis and myelination necessary for timely and robust action potential propagation. *In vitro* models have demonstrated that there is substantial ionic disruption at lower levels of axonal strain (Yuen et al., 2009), while at higher strains Na^+^ channel disruption leads to calcium influx and changes in the channel subunit distribution (Wolf et al., 2001; Iwata et al., 2004). These changes in the axons after injury could lead to a total disruption of signal transmission initially, and then compromised or delayed propagation over long periods of time due to compensatory channelopathies (Yuen et al., 2009).

Depolarization due to ionic imbalance could also underlie synaptic communication deficits, as the loss of driving force may affect the calcium influx necessary for synaptic release (Reeves et al., 2005; Goforth et al., 2011). Previous TBI models have demonstrated changes in axonal conduction in both myelinated and unmyelinated axons in the collosum (Reeves et al., 2000, 2005; Colley et al., 2010), as well as in presynaptic fiber volley amplitudes in various models, all of which could disrupt precise signal timing and integration of inputs (Norris and Scheff, 2009; Reeves et al., 2000; but see Cole et al., 2010). While understanding cellular mechanisms of altered action potential conduction velocities and neuronal firing properties links traumatic axonal injury to timing disruption in neuronal communication and may lead to secondary injury prevention strategies, it does not address how these abnormalities disrupt network level function leading to cognitive deficits.

### Global Functional Brain Networks are Altered Following TBI

At the global scale, non-invasive functional imaging such as functional magnetic resonance imaging (fMRI) is used to determine global or regional brain network differences between human TBI patients and healthy controls, which are then correlated to cognitive dysfunction. Structural imaging techniques such as DTI can delineate broadly the white matter patterns of injury and attempt to link them to both cognitive dysfunction (see above) as well as network abnormalities determined by fMRI (MacDonald et al., 2008; Palacios et al., 2012; Tang et al., 2012) and for review see Xiao et al. (2015). Recent advances in functional imaging analysis techniques have capitalized on the inherent fluctuations in regional brain activity during quiet rest to identify brain regions that fluctuate together (are “functionally connected”) and which become relatively deactivated when the brain engages in non-self-referential goal-directed tasks. This major network of activity has been called the default mode network (DMN), thought to represent an intrinsic core network in the absence of significant sensory input (Raichle, 2015). The DMN generally includes bilateral frontal, prefrontal and parietal regions as well as the cingulate, and has been identified in humans, non-human primates and rodents (Xiao et al., 2015). DMN activation, deactivation and connectivity have all been found to be altered in TBI patients compared to healthy controls and correlated with cognitive impairment (Bonnelle et al., 2011, 2012; Sharp et al., 2011; Palacios et al., 2012; Xiao et al., 2015). While these methods have been utilized to generate generalized hypotheses about connectivity and treatment after TBI (Ham and Sharp, 2012), they do not easily suggest any specific therapeutic targets as they lack a well-defined relationship to the presumed underlying abnormalities in neuronal communication.

### Understanding Network Timing Dysfunction May Lead to New Therapeutic Strategies

The predominant treatments that have been developed over the last 20 years for TBI have centered around sparing neurons from degeneration post injury, although recently attention has been paid to the axonal connections between neurons as well (Smith et al., 2013). However, the success of translation has been poor for most agents, and few were designed to modulate axonal dysfunction or its downstream effects. There are also currently no treatments for mild TBI that are considered effective, particularly for post-concussive symptoms. We propose a systems approach that lies between the micro scale of sodium channel disruption and the macro scale of global functional brain connectivity to address the following questions:

- How does pathological alteration in the sub-second timing of neuronal communication lead to impaired formation of “neuronal ensembles” (see below) as well as the local and global breakdown of interactions between ensembles?
- How does this network dysfunction lead to memory and executive function impairment seen after TBI?

We propose that hypotheses directed at understanding the timing relationships between networks of neurons at the millisecond level, and how disruption of this timing after TBI leads to cognitive impairment, may offer an important and largely unexplored pathway towards novel therapeutics aimed at restoring lost function via new electrophysiological targets. For example, animal models of TBI demonstrate a loss of theta frequency power in the hippocampus. Stimulation of medial septal projections to the hippocampus *within the theta frequency range, but not outside of it*, has been shown to recover behavior in these models (Lee et al., 2013, 2015). We propose to develop directed therapies that are predicated on a detailed understanding of the electrophysiological impairments following injury.

## Construction and Integration of Neuronal Ensembles Requires Precision Timing

### Neuronal Ensembles

Perception of sensory stimuli, short and long-term memory, and even habits are thought to be encoded and represented by distributed neural assemblies termed “ensembles” across the brain (Pennartz et al., 1994; Buzsáki and Chrobak, 1995; Sutherland and McNaughton, 2000; Buzsáki, 2004). Multi-unit recordings have recently identified the neuronal activity underlying many of these representations during awake behavior (Laubach et al., 2000; Hoffman and McNaughton, 2002; Barnes et al., 2005). Memory has long been described by Hebbian processes of changing connection strength between neurons, and more recently the “engram” or distributed neuronal memory trace associated with some aspects of memory has been causally demonstrated using optogenetic and other techniques (Hebb, 1949; Lashley, 1950; Liu et al., 2012; for review see Josselyn et al., 2015; Tonegawa et al., 2015).

Many interesting questions remain, however, including how shorter-term memory in the hippocampus is integrated with other memories and moved out to the cortex, and how this distributed memory is then represented in the cortex. How are spatial (across the brain or brain regions) and temporal (over time) representations of the above examples (perception, etc.) coordinated over such long distances? How is it that ensembles in the cortex receive discrete contextual information from groups of cells in the hippocampus such as “place” cells? One mechanism for coordinating different regions of the brain and transmitting information between them is an oscillation, a foundational concept in neuroscience and neurology whose mechanistic role in these processes has been debated since its discovery. Another concept for distributed representations of information in the cortex are “syn-fire chains”, a model that demonstrated that a synchronously firing series of neurons could propagate (or transmit) information through noisy layers (Abeles et al., 1993; Aertsen et al., 1996; Diesmann et al., 1999). These concepts are not mutually exclusive, but both require exquisite timing and ensembles of coordinated cell firing. These ensembles must be coordinated across great physical lengths, potentially including across hemispheres, suggesting that traumatic injury to the conduits of this precise information exchange could lead to significant impairment.

### Oscillations and Entrainment are Fundamental Mechanisms of Neuronal Communication at Multiple Scales

Synaptic communication between neurons within local ensembles, or across long distances between ensembles, requires the conduction of current along axons and the release and binding of neurotransmitters at synaptic junctions leading to current flow. If sufficient coordinated input current flows into an ensemble of neurons, that neuronal ensemble may alter its output (firing pattern) to be more synchronous with the dominant currents it is receiving. A single neuron has many sources of current, both excitatory (depolarizing) and inhibitory (hyperpolarizing) from many local and distant brain regions that are integrated over time. One can envision that if one or more afferent white matter tracts have been injured, the inflow of synaptic currents to a given neuronal ensemble may no longer be sufficiently coordinated to maintain its precisely timed output, leading to functional impairment.

The combined current influx into a localized brain region can be measured as the *local field potential (LFP)*. Cortical LFP can be measured with scalp EEG, subdural electrode grids, or with individual electrodes placed in the brain. The LFP of deeper structures (in the hippocampus, for example) requires depth electrodes that penetrate the structure. Patterns of oscillations in the LFP within different frequency bands have been observed in all mammalian species studied and are linked to behavioral states (Buzsáki et al., 2012). For example, in primates, alpha (8–12 Hz) oscillations are observed to dominate the occipital lobe during quiet rest with the eyes closed (Jurko et al., 1974), a rhythm proposed to arise from thalamocortical projections in the absence of sensory input (Buzsáki and Watson, 2012). Theta oscillations (4–10 Hz in rats) in the hippocampus have been observed to dominate during navigation, memory tasks, and rapid eye movement (REM) sleep (Buzsáki and Moser, 2013) and appear to depend on projections from the medial septum (Buzsáki, 2002; see below).

LFP oscillations represent dominant, coordinated inflow of current into the region being measured, not necessarily the pattern of firing of the local neurons (Buzsáki et al., 2012). Only if those inputs are strong enough and sufficiently synchronized will they influence when the local neurons fire. For example, when a particular oscillating input dominates current inflow, local neurons may preferentially fire more often during a particular phase of that oscillation, called *entrainment*. There may be multiple local and distant sources of oscillatory input at different frequencies, all of which are influencing the timing of local neuronal firing. In this way the resulting pattern of firing is an integration of the influences of many disparate brain regions, as well as the local connections in the ensemble, in combination with the cell’s intrinsic properties. Furthermore, this varies over time: different behavioral states or actions may be characterized by the dominance of one oscillation over others at any given moment. As described below, this is the case in the hippocampus. The patterned influence of current inflow from many regions on the subsequent firing pattern of a neuronal ensemble is a major proposed mechanism by which information is coordinated and transmitted between brain regions, and one that relies critically on precise, sub-second timing.

### Spike Timing Dependent Plasticity

Another axonal timing dependent phenomena, spike-timing dependent plasticity (STDP), is a well described phenomenon whereby neurons that fire reinforce the synapses on their own dendrites using back-propagating actions potentials (Tritsch and Sabatini, 2012). In this manner, those inputs onto the dendrites that collectively led to the firing of a neuron are strengthened. The timing of the dendritic inputs and the timing of the resulting firing of the neuron have been proposed as one basis for memory formation in the hippocampus. When specific axons are temporarily or permanently dysfunctional, this may drastically affect the ability of the neurons to integrate incoming information over time and appropriately reinforce the relevant synaptic inputs. At another level, if this were to occur to numerous cells at the same time, the loss of the ensemble activity that represents a “memory” may be temporarily or permanently disrupted, as would the ability to encode new memories.

## Timing Deficits in Hippocampal Networks Hypothesized as Mechanism Underlying Memory and Executive Dysfunction After TBI: From Local to Global

The hippocampus is one of the most studied brain regions in both TBI and memory due to its central role in short-term and spatial memory and its demonstrated vulnerability during TBI. Hippocampal networks are TBI-relevant examples of the processes described above, allowing us to develop specific hypotheses about hippocampal network dysfunction following TBI at both local and global scales that can be tested using a systems neuroscience approach. Beyond the hippocampus proper, wider hippocampal networks that include reciprocal connections to entorhinal cortex, striatum, septal region, amygdala and PFC are known to be important for memory and executive functioning. Some of these connections may be preferentially injured following TBI, as discussed above (see “Memory and Executive Function are Persistent Cognitive Deficits after TBI” section). Further, much is known about the role of oscillatory activity and precision timing within hippocampal networks as they relate to memory formation, consolidation and executive function.

### Two Complementary Network States Characterize the Awake Hippocampus and May Become Dysfunctional After TBI

In the awake animal, two dominant electrophysiological states have been described in the hippocampus. In the rodent, theta frequency band oscillations (approximately 4–10 Hz) dominate the hippocampal LFP during spatial exploratory activity, memory tasks, and REM sleep (Buzsáki, 2002). Immobility, eating, grooming, and non-REM slow wave sleep are dominated by slower, large amplitude irregular LFP activity punctuated by high amplitude spikes called sharp waves (Buzsáki, 2015). It is thought that during exploratory, theta-dominant activity, sequences of “events” are linked to form a memory. These temporally linked events could be a series of locations visited by a rat in a box that are integrated to form an internal map of the rat’s environment, or as proposed by Buzsáki and Moser (2013) they could be more complex cognitive episodes combined to form an episodic memory, such as the steps in tying one’s shoe (Buzsáki, 2005). In contrast, there is evidence that during sharp wave dominant states of immobility or non-REM sleep, specific sequences of neuronal firing representing these episodic memories, are “re-played” in a temporally compressed manner and may be undergoing some form of consolidation and redistribution to other connected areas of the brain (Lee and Wilson, 2002; Foster and Wilson, 2006; Diba and Buzsáki, 2007; Girardeau et al., 2009; and for review see Eichenbaum, 2000; Carr et al., 2011). The coincidence of memory dysfunction and sleep disturbance after TBI is intriguingly suggestive of a failure of one or both of these hippocampal states, or the balance between them.

#### The Exploratory State: Place Cells, Theta Oscillations and Memory Formation

Perhaps the most well-studied neuronal function to date is that of the hippocampal place cell. First described in rats by O’Keefe and Dostrovsky (1971), place cells are pyramidal cells in the hippocampus that modulate their firing rate based on the position of the animal within its spatial environment (O’Keefe and Dostrovsky, 1971). A given ensemble of place cells will increase their firing rate if the animal traverses one or more locations within its environment, termed the cells’ place field. In this way a map of an animal’s environment and its current position therein is maintained by a collection of neuronal ensembles. As an animal moves along a particular path in its environment, a sequence of place cell ensembles will fire in an order that represents the path taken. A memory of the animal’s traveled path is therefore formed, represented as a temporal sequence of place cell ensemble firings. This basic mechanism can be extended and applied to other episodic memories, such as tying a shoe (Buzsáki and Moser, 2013).

Although the mechanisms are poorly understood, it is clear that the formation of a place cell is dependent on coordinated input into the hippocampus from “grid cells” found in the entorhinal cortex, via direct and indirect projections to the CA1 hippocampal subfield along the perforant and temporoammonic pathways. Lesions to the direct projections from entorhinal cortex to CA1 result in impaired place cell formation (Tonegawa and McHugh, 2008), and inducing trauma to the perforant pathway leads to impaired spatial memory in rodents (Skelton, 1998; Perederiy and Westbrook, 2013). Furthermore, DTI evidence of perforant pathway injury has been correlated with memory impairment in patients with severe TBI (Christidi et al., 2011). We therefore hypothesize that axonal injury to perforant pathway projections would lead to impaired place cell formation and spatial memory deficits after TBI (McNaughton et al., 2006a).

A critical property of hippocampal place cells is that they are synchronized, or entrained, to the hippocampal theta oscillation during exploration. The phase of the theta cycle at which a place cell fires also changes in a predictable way as the animal moves through the place field of that cell, termed “phase precession” (O’Keefe and Recce, 1993). It is thought that theta oscillations in the hippocampus provide a structure into which neuronal firing sequences such as place cell ensembles are organized, compress the time scale of ensemble sequences such that synaptic plasticity can take place, and provide a structure for synchronization with other brain regions (Buzsáki and Moser, 2013). It is therefore critical that the timing of synaptic currents into place cell ensembles is maintained with precision. Studies in animals and humans have shown that learning trials in a memory task occurring during periods of time when theta power is increased in the hippocampus lead to improved memory performance (Seager et al., 2002; Sederberg et al., 2003; Merkow et al., 2014). Additionally, there is early evidence that hippocampal theta power is reduced in the lateral fluid percussion model of TBI and that this may correspond to reduced memory performance (see below; Fedor et al., 2010; Lee et al., 2013, 2015; Paterno et al., 2016).

The origin of the hippocampal theta oscillation is complex, but it is clear that it is dependent on extra-hippocampal synaptic inputs. A key structure with reciprocal GABAergic connections to the hippocampus via the fornix is the medial septum/diagonal band of Broca (MS-DBB; Freund and Antal, 1988; Tóth et al., 1993). Rhythmically firing cells in the MS-DBB are entrained to hippocampal theta oscillations and persist in their rhythmicity even when theta oscillations no longer dominate the hippocampus (Petsche et al., 1962). Both physical and drug-induced lesions of the MS-DBB, as well as lesions of the septohippocampal projections of the fimbria-fornix, abolish theta oscillations in the hippocampus (Petsche et al., 1962; Stumpf et al., 1962; Buzsáki et al., 1983; Buzsáki, 2002; McNaughton et al., 2006b). This and other evidence led to the original conceptualization of the MS-DBB as the rhythmic “pacemaker” of hippocampal theta oscillations (Buzsáki et al., 1983), although it is now believed that theta arises from reciprocal inhibitory connections between the MS-DBB and hippocampus along with cholinergic septohippocampal tone, entorhinal cortical inputs, recurrent collateral projections from CA3, and intrinsic properties of intra-hippocampal interneuron inhibitory circuits (Dragoi et al., 1999; Buzsáki, 2002; Vandecasteele et al., 2014) Interestingly, it has recently been demonstrated that bidirectional long-range GABAergic projections exist between the entorhinal cortex and the hippocampus and contribute to theta generation, suggesting another vulnerable pathway for theta disruption via axonal injury (Melzer et al., 2012).

Not only are intact connections between the hippocampus and MS-DBB necessary for hippocampal theta oscillation generation, but their loss and subsequent functional restoration with electrical stimulation are linked to memory performance deficits and subsequent recovery, respectively (McNaughton et al., 2006b). There is also early evidence for the relevance of this circuitry dynamic in TBI models. Lee et al. (2013, 2015) conducted a series of experiments in rats after lateral fluid percussion injury demonstrating both a reduction in performance on the Barnes Maze (a rodent spatial memory task) and a reduction in hippocampal theta power, as well as the temporary restoration of theta power and maze performance with theta frequency stimulation of the MS-DBB. Furthermore, while theta power in the hippocampus was reduced after TBI it remained unchanged in the MS-DBB, as did theta coherence between MS-DBB and the hippocampus, suggesting a specific disruption of the septohippocampal circuitry (Lee et al., 2015). This reduction in theta power after fluid percussion injury has also been described in a mouse model that demonstrated sleep disruption after TBI (Lim et al., 2013).

We therefore hypothesize that reductions in theta hippocampal power observed post-TBI may be related to axonal injury of numerous pathways projecting into the hippocampus, in addition to hippocamposeptal reciprocal projections. Furthermore, reductions in theta power may lead to impaired entrainment and therefore impaired temporal organization of place cell ensembles, or a reduction in phase precession, which ultimately leads to impaired memory formation and impaired spatial navigation.

#### Place Cells and Cross Frequency Coupling

The synchronization between oscillations of different frequencies within and between brain regions is termed cross-frequency coupling (CFC) and has recently received a great deal of attention (for review see Hyafil et al., 2015). The representation of multiple items during working memory, the parsing out of various stimuli, and long-distance communication have all been linked to CFC (Hyafil et al., 2015). One form of CFC, phase-phase coupling, may be particularly sensitive to timing disruptions and may therefore be affected by TBI and axonal injury. Theta and gamma (~40 Hz) oscillations in particular have been demonstrated to couple in this manner, and have been proposed to form a “code” whereby multiple items can be represented in working memory in an ordered fashion (Lisman and Buzsáki, 2008; Lisman and Jensen, 2013).

In this model, not only do place cell ensembles (or cell ensembles representing any memory item) fire in temporal sequence entrained to the theta oscillation (see above), but each individual ensemble is locked to a cycle of the gamma oscillation. Several gamma cycles are, in turn, phase-locked to a single theta cycle. In this way gamma oscillations serve to organize neuronal ensembles representing individual memory items, while the phase relationship to theta oscillations serves to organize these memory items into a defined temporal sequence, forming the basis of episodic memory and potentially serving as the structure that allows distribution across brain regions (Lisman and Jensen, 2013). Theta-gamma CFC has been implicated in both working and long-term memory operations in rodents and humans, and in both hippocampus and cortex (Canolty et al., 2006; Tort et al., 2009; Maris et al., 2011; Friese et al., 2013). Theta-gamma CFC has recently been demonstrated to increase in healthy human volunteers during an n-back task that required ordering of information in working memory (Rajji et al., 2016). We hypothesize that timing delays due to axonal injury and dysfunction may lead to the disruption of the appropriate encoding of this ordered information via CFC, and that this mechanism may underlie aspects of TBI-related deficits in working memory.

The source of gamma oscillations is thought to be intrinsic properties of GABAergic interneurons and therefore a “locally generated” phenomenon (Hyafil et al., 2015). Interestingly, there is evidence to suggest that hippocampal interneurons may be preferentially injured following TBI (Lowenstein et al., 1992; Almeida-Suhett et al., 2015). We therefore hypothesize that hippocampal gamma oscillation power may also be reduced following TBI, leading to impaired theta-gamma coupling and a reduction in the temporal structure necessary for the formation of episodic memory.

#### The Sharp Wave-Ripple State: the “Deafferented Hippocampus”

The theta dominant state of the hippocampus depends on integrating input from many regions and is active during spatial exploration and REM sleep. In contrast, the sharp wave-ripple (SPW-R) state of the hippocampus is thought to be internally generated, dominant during quiet rest, eating, grooming, and deep, non-REM sleep (Buzsáki, 2015). Indeed, recordings from fetal hippocampi that have been implanted into adult rats (and therefore deprived of natural inputs from other brain regions) reveal a dominance of sharp waves and the synchronization of pyramidal neuronal firing to them, persisting even during spatial exploratory activity that normally suppresses sharp waves in favor of theta dominance (Buzsáki et al., 1987a,b). Electrophysiologically, the SPW-R state occurs when hippocampal afferent inputs are reduced (Buzsáki, 2015). It is dominated by irregular activity punctuated by large amplitude sharp waves 40–100 ms in duration, thought to originate from the CA3 region. Brief episodes of fast oscillations (150–240 Hz) occur in the CA1 region at the peaks of the sharp waves, termed ripples (Buzsáki, 1986). These ripples are thought to be generated by fast local interneuron synaptic inhibition onto CA1 pyramidal cells in response to Schaffer collateral synaptic input from CA3. This inhibition leads to strong entrainment of the pyramidal cells to the ripple (Buzsáki, 2015).

The functional significance of the hippocampal SPW-R state is not well-understood, but there is increasing evidence that it may play a critical role in memory consolidation and re-distribution to extra-hippocampal brain regions (Girardeau et al., 2009; Buzsáki, 2015). Remarkably, it has been shown in rats that the specific temporal sequence of place cell firing that occurs when a rat moves through its environment, initially synchronized to theta oscillations (see above), is replayed in exact reverse sequence immediately after the spatial experience (Foster and Wilson, 2006; Diba and Buzsáki, 2007; Girardeau et al., 2009) and during slow wave sleep (Lee and Wilson, 2002), as well as in forward sequence immediately prior to the repetition of that spatial experience (Diba and Buzsáki, 2007). However, these place cell sequence replays occur during ripple oscillations, temporally compressed approximately twenty-fold, in bursts of approximately 100 ms (Lee and Wilson, 2002). This precisely-timed process is thought to play a role in the consolidation and possibly distribution of episodic memory to a wider neocortical network (Logothetis et al., 2012), particularly as ripples have been detected in other brain regions, and as the artificial elimination of sharp wave ripples during post-training sleep leads to impairment of subsequent memory performance in rats (Wierzynski et al., 2009; Girardeau et al., 2009).

The consequences of TBI on the SPW-R state are potentially numerous. First, we hypothesize that axonal injury to intra-hippocampal projections such as the Schaffer collateral system from CA3 to CA1, or the disruption of interneuronal circuitry (particularly in light of evidence for preferential loss of hippocampal interneurons; Lowenstein et al., 1992; Almeida-Suhett et al., 2015), may lead to a reduction in ripple generation and/or an impaired ability for place cells to entrain to them. This may lead to memory consolidation impairment. Second, we hypothesize that injury to efferent axonal projections from the hippocampus (via the fornix, for example) may disrupt the transmission of ripple-associated relay events to the neocortex, leading to impairment of memory re-distribution. Third, we hypothesize that widespread loss of synaptic input into the hippocampus (e.g., entorhinal projections) due to axonal injury could inappropriately bias the hippocampus towards its deafferented, synchronous state, the SPW-R state, which is further supported by a recent set of experiments demonstrating pathological high frequency activity after perforant path disruption (Ortiz and Gutiérrez, 2015). This could potentially cause widespread memory formation and consolidation difficulties, sleep cycle disturbance, and epileptiform activity, all symptoms classically observed after TBI. A recent study combining single cell recordings in the hippocampus with resting state fMRI in non-human primates demonstrated a consistent increase in DMN activity after hippocampal ripple events (Kaplan et al., 2016). This finding coupled with previous work demonstrating inappropriately persistent DMN activity during cognitive tasks in patients with TBI (Bonnelle et al., 2012) suggests a global network related to an internal hippocampal SPW-R state that is unable to disengage appropriately with cognitive demand.

Finally, as discussed, the hippocampal SPW-R state is internally generated and thought to be the most synchronous state of the hippocampus (Buzsáki, 2015), which may be of particular relevance to post-traumatic epilepsy. Ripples share some characteristics with pathological high frequency oscillations (pHFOs) seen during interictal periods of epilepsy that are predictive of seizure onset (a hyper-synchronous state; Bragin et al., 2010). Others have proposed that pHFOs may be generated by the disruption of firing reliability associated with the SPW-R, leading to offset ripple oscillations (Foffani et al., 2007; Ibarz et al., 2010). This suggests that epileptogenesis could result in part from a hippocampal network in an abnormally persistent SPW-R state, or one with disrupted timing patterns leading to pathological oscillations.

### Global Hippocampal Network Dysfunction After TBI

The hippocampus is part of the larger limbic system, including the PFC, the amygdala and the striatum. There are numerous examples in the limbic system of how oscillations in one region can influence neuronal ensembles in another, suggesting that this system is highly interconnected by axonal pathways carrying timing dependent information. We hypothesize that cognitive processes subserved by these interconnected pathways could become impaired after TBI.

#### Hippocampal Prefrontal Cortical Synchronization and Working Memory

Projections from the hippocampus to the PFC and the striatum carry output that is entrained to hippocampal theta oscillations. This theta-entrained output leads to the entrainment of target (i.e., PFC and striatal) neurons to hippocampal theta (Berke et al., 2004; Jones and Wilson, 2005a). In rodent models theta oscillations in the PFC in addition to the hippocampus are important for spatial navigation and spatial working memory (Jones and Wilson, 2005b; Benchenane et al., 2010; Spellman et al., 2015). Jones and Wilson demonstrated that when rats must make a choice about the correct location of a food reward using working memory, two things happen between the PFC and the hippocampus: first, the normally distinct theta oscillations of each structure synchronize, and second, the firing of PFC neurons becomes entrained to the theta oscillations in the hippocampus (Jones and Wilson, 2005b). Importantly, this synchronization and entrainment was only observed when the rat made the correct choice and progressively became stronger up to the point of decision. Along with rodent studies supporting the role of theta synchronization between hippocampus and PFC during working memory, recent advances in human magnetoencephalography (MEG) using source localization have demonstrated that temporal lobe theta amplitude successfully predicts integration of memories, and that this involves increased hippocampal-PFC theta coupling (Backus et al., 2016). In light of human structural evidence that PFC-hippocampal white matter tracts may be preferentially impaired following TBI (see “Memory and Executive Function are Persistent Cognitive Deficits after TBI” Section), these studies lead us to hypothesize that axonal injury following TBI may lead to impairments in working memory due to an inability of the hippocampus and PFC to couple at the appropriate time via theta oscillations, either due to latency or conduction issues along the pathways between these structures, or due to a primary reduction in hippocampal theta power, or both. We therefore predict reduced coupling of hippocampal and PFC theta oscillations, and reduced context appropriate entrainment of PFC neurons to hippocampal theta oscillations post-TBI.

#### Hippocampal, Prefrontal Cortical and Amygdalar Synchronization and Affective Memory: TBI and Post-Traumatic Stress Disorder Comorbidity?

Recent work from a number of laboratories has examined the interactions between the limbic structures known to be involved in another type of learning called “fear conditioning”. This classical conditioning paradigm is performed by pairing a cue to an aversive stimulus, after which presentation of the cue without the stimulus then elicits a fear response (Maren, 2001). This paradigm has been described as a model of post-traumatic stress disorder (PTSD), as the effects of this pairing are long lasting, and can only be extinguished by numerous presentations of the cue without the stimulus. The strongest link between limbic areas during acquisition and extinction of fear memory appears to be between the PFC and amygdala, as increases in both theta and gamma oscillation synchrony have been reported within and between these structures during various stages of a fear conditioning task (Likhtik et al., 2014; Stujenske et al., 2014). Consolidation of fear memory involves theta oscillatory synchronization between the hippocampus and amygdala, which appears in the days after training (Seidenbecher et al., 2003). In contrast, oscillatory synchrony occurs between all three structures during REM sleep after training, which has been suggested as a predominant time when memory is consolidated, suggesting a specific role for oscillation synchronization in the consolidation of fear memory (Popa et al., 2010). While the mechanisms underlying this long distance synchronization remain to be elucidated, it is clear that behavioral state and learning changes are correlated with changes in oscillations shared by or propagated between these limbic regions during fear and anxiety associated behavior.

PTSD is a frequent comorbidity with TBI in the military population, with almost 35% of mild TBI exposed Veterans reporting qualifying symptoms associated with their service in theater (Galarneau et al., 2008; Stein and McAllister, 2009). However, a great deal of controversy remains over whether mild TBI contributes to the susceptibility for PTSD, or whether TBI mechanistically underlies some aspects of presenting PTSD symptoms (Hoge et al., 2008; Stein and McAllister, 2009). The presenting symptomatology of PTSD (i.e., emotion dysregulation, sleep and cognitive deficits) may have an underlying basis in the biomechanical disruption by TBI of the pathways coordinating limbic regions and their oscillatory network interactions described above. TBI may also disrupt the substrates for the consolidation processes necessary for the predominant treatment for PTSD, extinction therapy.

## A Systems Neuroscience Approach to TBI

All cognition and memory is formed from neural ensemble coding, which has a spatial and temporal component distributed throughout the brain and that is linked via axonal connections. TBI has the potential to disrupt the spatiotemporal interaction between ensembles due to changes in axonal latency, via axonal loss due to dysfunction or degeneration, leading to changes in synaptic and intrinsic neuronal properties. As we have described, timing disruptions in hippocampal networks from the local to the global level may both explain some of the persistent symptoms following TBI, including memory and executive function impairment, and suggest therapeutic strategies for intervention and recovery of lost function.

We have argued that axonal injury to pathways within the hippocampus such as Schaffer collaterals, as well as pathways that connect the hippocampus to other larger networks such as the perforant pathway, the fornix, and the cingulate bundle (connecting the hippocampus to the PFC) leads to timing delays in neuronal communication particularly relevant to TBI symptoms. Several overarching hypotheses emerge:

- TBI induced axonal injury results in loss of, or derangement in synchronization within intra-hippocampal connections. This may affect proper hippocampal oscillatory function, including the temporal organization of neuronal ensembles involved in memory construction (e.g., place cells).
- TBI induced axonal injury results in loss of, or pathological delay in synaptic input into the hippocampus. This may affect organizing oscillations from extra-hippocampal sources (e.g., theta), proper formation of neuronal ensembles encoding spatial or episodic memories, and the coordination of both.
- TBI induced axonal injury to long-range connections between the hippocampus and other distant structures may impair coordinated oscillatory activity between these regions and adversely affect executive functions such as working memory, or affective processes such as fear conditioning.

### Neuromodulation Strategies Based on Neuronal Timing Disruptions

Neuromodulation strategies arising from these hypotheses would not only reveal anatomical targets for stimulation or inhibition, but also suggest timing frequency parameters for such interventions. For example, much of the history of deep brain stimulation for movement and psychiatric disorders, a classic neuromodulation therapy, has focused on anatomical targets and has largely employed an empirical method for determining stimulation parameters (Lozano and Lipsman, 2013). We propose a fundamentally different strategy, one that employs an understanding of the timing deficits within neuronal circuits and then seeks to restore them, or compensate for them. For example, one might envision providing electrode stimulation of the septo-hippocampal pathway at a theta frequency, either by medial septal stimulation, or direct fornix stimulation, to restore organizing theta oscillations in the hippocampus to support memory function, a strategy that has already been employed in rodent models (Lee et al., 2013, 2015). This could be further developed into a closed loop system that might limit theta stimulation to periods of low SPW-R activity, or in response to increased entorhinal cortical activity.

An approach based on dysfunctional neuronal timing may also lead to molecular therapeutic strategies. It has been proposed that myelination and related latency, or signal conduction speed, may in fact be a plastic phenomena, suggesting that there may be some control exerted by the neuron over how fast a signal can be propagated to its targets (Pajevic et al., 2014). If demonstrated conclusively, this could be a mechanism for repair after TBI that is already being utilized by the brain after injury, and one that could be exploited for therapeutic applications should there be widespread latency delays. Latency delays have been demonstrated in human TBI utilizing evoked potentials, and have been correlated with outcomes in some studies, but whether these latency changes in evoked responses are diagnostic remains controversial, and they have been used predominantly in moderate to severe TBI (Rappaport et al., 1991; Keren et al., 1991; Lew et al., 2004; Morgalla and Tatagiba, 2014). These results have not been reproduced in animal models to date, presumably due to the axonal distances required to demonstrate a detectable change in latency. These should be undertaken in large animal models in order to measure latency effects and test therapeutic ideas for restoring axonal function.

### Animal Models of Axonal Injury, Human Confirmation of Network Dynamics

Animal models of pure diffuse axonal injury remain rare due to the difficulty in generating the biomechanical forces necessary to induce inertial injuries. However, existing inertial injury models in gyrencephalic species have demonstrated similar axonal pathology to the patterns and distribution of human diffuse axonal injury (Smith et al., 1997). At lower levels of rotation, presumed to model mild TBI, axonal pathology was still widely detected (Browne et al., 2011). However, although not models of *inertial* injury, axonal loss and injury also occur in many rodent TBI models, suggesting that they can be utilized to test selected hypotheses presented here in affected pathways (Johnson et al., 2015). Similarly, a significant limitation of our understanding of network oscillations and entrainment is that it is also based on rodent studies, although many of the mechanisms we describe have been observed in humans and non-human primates, as noted in previous sections. Thus, it is critical that mechanisms of network synchrony are verified in higher-order species, including humans, and that methods are developed for electrophysiological investigations in gyrencephalic animal models of traumatic axonal injury more representative of the human condition. Developing these strategies and the detailed understanding that will support them requires the tools of systems neuroscience, including electrophysiological analysis of both single neuron behavior and circuitry changes after TBI in awake animals, as well as computational modeling to develop more specific experimental predictions. As more is revealed about how regional neuronal interactions are disrupted via axonal injury, treatments directly targeting these dysfunctional pathways using neuromodulation can be developed.

## Conclusion

All cognition and memory is formed from neural ensemble coding, which has a spatial and temporal component distributed throughout the brain and that is linked via axonal connections. TBI has the potential to disrupt the spatiotemporal interaction between ensembles due to changes in axonal latency, or via axonal loss due to dysfunction or degeneration leading to changes in synaptic and intrinsic neuronal properties. In order to better understand these changes in the brain post injury, we must begin to use the tools of systems neuroscience to “listen to the neuronal ensemble” as well as to begin to modulate these connections so that we may better understand how to develop both neuromodulatory and molecular therapies for this condition.

## Author Contributions

JAW and PFK conceived of and wrote the article.

## Funding

This work was made possible due to financial support provided by the Department of Veterans Affairs Rehabilitation Research and Development (Merit Review Award #B1097-I, Career Development Award #IK2-RX001479), the CURE Taking Flight Award, and the Codman Neurotrauma Award.

## Conflict of Interest Statement

The authors declare that the research was conducted in the absence of any commercial or financial relationships that could be construed as a potential conflict of interest.
